# Screening, isolation, identification and evaluation of bacteria with probiotic potential from traditional palmyra palm nectar

**DOI:** 10.3389/fcimb.2025.1685639

**Published:** 2025-11-03

**Authors:** Raman Kanimozhi, Ravichellam Sangavi, Nambiraman Malligarjunan, Shanmugaraj Gowrishankar

**Affiliations:** Department of Biotechnology, Alagappa University, Karaikudi, India

**Keywords:** probiotics, palm nectar, β-galactosidase production, gastrointestinal conditions, eps production, anti-oxidant activity and indigenous fermented foods.

## Abstract

**Introduction:**

Traditional fermented foods are rich reservoirs of probiotic microorganisms, yet several remain scientifically underexplored. The current research focused on the screening, isolation, identification, and assessment of potential probiotic isolates exhibiting β-galactosidase activity from naturally fermented Palmyra palm (*Borassus flabellifer*) nectar, a culturally significant, traditional beverage consumed in India.

**Methods:**

A total of 80 bacterial isolates were obtained under aseptic conditions and screened through cultural, microscopic, and biochemical analyses. Fifty-six Gram-positive, catalase-negative isolates were shortlisted for probiotic evaluation. Selected isolates were assessed for simulated gastrointestinal conditions, cell surface properties (auto-aggregation, hydrophobicity, co-aggregation and Biofilm production), β-galactosidase and exopolysaccharide production, antioxidant activity, antibiotic susceptibility, and safety through hemolysis and DNase activity.

**Results:**

Seventeen isolates exhibited desirable adhesion-related traits, of which seven strains demonstrated superior probiotic potential. These strains tolerated acidic and bile conditions, produced high levels of exopolysaccharides (573-785 mg/L) and β-galactosidase (110.25-221.09 U/mL), and showed significant cell surface hydrophobicity (35.87-69.93%), auto-aggregation (59.29-82.76%), and co-aggregation with pathogens *Salmonella* Typhi (MTCC 733) 46.58 - 70.87% and *S. flexneri* (ATCC 12022) 53.45 - 78.85%. They also exhibited substantial hydroxyl radical scavenging activity (57.68-70.66%) and were safe and antibiotic-susceptible.

**Discussion:**

The findings highlight the probiotic potential and functional attributes of Palmyra nectar - derived bacteria. Their ability to survive gut-like conditions, hydrolyze lactose, adhere to intestinal mucosa, and provide antioxidant benefits supports their application in functional foods and nutraceuticals aimed at improving gut health and lactose digestion.

## 1 Introduction

The resurgence of interest in traditional fermented foods stems from their long-standing use in diverse cultures and their growing recognition as rich reservoirs of health-promoting microorganisms. Among these, probiotics, defined as “live microorganisms which, when administered in adequate amounts, confer a health benefit on the host” (Joint FAO/WHO Expert Committee on Food Additives. Meeting, & World Health Organization, 2006) have gained considerable attention for their ability to enhance gut health, modulate immunity, and aid in the management of metabolic and inflammatory disorders (Hill et al., 2014; Markowiak and Śliżewska, 2017). Probiotic organisms are widely acknowledged as Generally Recognized as Safe (GRAS) owing to their synthesis of bioactive compounds and well-documented health-promoting properties (Han et al., 2017). Various probiotic strains act as valuable sources of bioactive metabolites, which contribute significantly to genomic integrity and intercellular signaling, highlighting their potential for incorporation into functional foods and human diets (Pihurov et al., 2021).

Traditionally fermented plant-based beverages represent an underexplored niche for probiotic discovery (Lamba et al., 2019). They harbor diverse microbial communities that have adapted to unique ecological niches and selective pressures, often resulting in the evolution of strains with distinctive probiotic and functional properties (Tamang et al., 2020). While significant progress has been made in characterizing probiotics from dairy-based fermentations, plant-origin probiotics, particularly those from culturally significant fermented products, remain relatively under-investigated (Nandha and Shukla, 2023).

India’s rich biodiversity and ethnoculinary traditions offer a vast landscape for microbial bioprospecting (Tamang et al., 2021). Among traditional beverages, palmyra palm nectar, locally known as Pathaneer in Tamil Nadu, is a naturally fermenting sap extracted from the inflorescence of *B. flabellifer* Linn., a culturally significant palm species prevalent in South and Southeast Asia. Fresh Pathaneer is a mildly sweet, translucent liquid with a near-neutral pH (6.0–7.0), rich in carbohydrates, amino acids, vitamins, organic acids, and polyphenols (Lasekan and Abbas, 2010; DebMandal and Mandal, 2011). When collected under hygienic conditions and preserved at low temperatures, the nectar undergoes natural fermentation, primarily facilitated by native microbiota, majorly lactic acid bacteria (LAB) and acetic acid bacteria.

Traditionally, fermented Pathaneer has been utilized in Ayurvedic medicine for its reputed cooling properties, as well as its diuretic and digestive benefits. Recent studies have reported that LAB strains isolated from similar palm-based saps such as coconut Neera exhibit antimicrobial, antioxidative, and exopolysaccharide-producing abilities, validating their probiotic potential (Somashekaraiah et al., 2019; Thakur et al., 2021). These outcomes underscore the potential of such strains for use in food biopreservation and as starter cultures in regulated fermentation processes. As a result, there has been growing scientific interest in isolating and characterizing probiotics from diverse traditional fermented foods and products (Alonso et al., 2019).

Fermented palm nectar is a relatively untapped source for exploring exopolysaccharide (EPS) production and β-galactosidase activity. The commercial interest in β-galactosidase-producing probiotics has grown due to their potential application in managing lactose intolerance (Srinivash et al., 2023). Probiotic strains are often characterized by their ability to withstand harsh gastrointestinal conditions, including low pH, bile salts, and digestive enzymes. In addition, their functional efficacy is evaluated through properties such as co-aggregation, auto-aggregation, cell surface hydrophobicity, antioxidant function, adhesion to intestinal epithelial cells, possession of inhibitory effects towards enteric pathogens, and immunomodulatory potential (Saadat et al., 2019).

Under these circumstances, the study aimed to bioprospect and characterize β-galactosidase-producing bacteria from naturally fermented palmyra palm nectar collected from the southern districts of Tamil Nadu. This investigation aimed to (i) isolate and identify strains with probiotic traits, (ii) evaluate their functional properties, including EPS and β-galactosidase production, antioxidative capacity, and pathogen antagonism, and (iii) assess their tolerance to simulated gastrointestinal (GI) conditions. Through this work, we aim to uncover novel microbial candidates for application in functional food development, gut health management, and dairy fermentation, thereby contributing to the valorization of an indigenous and culturally relevant bioresource.

## 2 Materials and methods

### 2.1 Collection of samples

Fifteen freshly collected samples of palm nectar were retrieved from *Borassus flabellifer* trees, sourced from a traditional nectar tapper operating in a local farm located in Devakottai, Sivagangai district, Tamil Nadu, India. The nectar is naturally fermented under ambient conditions by traditional practices; the exact temperature and duration of fermentation were not controlled. To ensure sample integrity, they were transported aseptically and maintained at 4 °C until further analysis.

### 2.2 Selection and purification of microbial isolates

1 mL of every fermented culture was inoculated into 9 mL of De Man Rogosa Sharpe (MRS; Hi-Media Laboratories Pvt. Ltd.) broth and maintained at 37 °C for 48h to allow enrichment. Following incubation, serial dilutions ranging from 10^-^¹ to 10^-7^ were prepared using phosphate buffered saline (PBS). Aliquots from appropriate dilutions were plated and incubated at 37 °C for an additional 48 h (Krishnamoorthy et al., 2018). Colonies displaying diverse morphological traits were carefully picked and subjected to repeated subculturing on MRS agar across three successive transfers to ensure purity and stability of the isolates.

### 2.3 Bacterial strains and culture conditions

Five pathogenic indicator strains were selected for the assessment of antimicrobial assay. *Salmonella* Typhi (MTCC 733), *Shigella flexneri* (ATCC 12022), *Shigella sonneii* (ATCC 25931), *Klebsiella pneumoniae* (ATCC 700603), *Escherichia coli* (ATCC 49106). All bacterial strains were cultivated in Luria-Bertani (LB) broth (Hi-Media Laboratories Pvt. Ltd.) at 37 °C for 24 h.

### 2.4 Phenotypic and biochemical profiling

Morphological analysis was performed using Gram staining, while biochemical profiling included catalase testing and evaluation of carbohydrate fermentation patterns using the HiCarbo™ kit. Strains showing catalase negativity and displaying Gram-positive morphology - characteristic features of lactic acid bacteria - were identified as potential probiotic candidates.

### 2.5 Functional assessment of probiotic traits

#### 2.5.1 Resistance of probiotics to stimulated GI conditions

##### 2.5.1.1 Tolerance to acidic and bile conditions

Acid and bile resistance of the isolates was evaluated based on the procedure described by Bhushan et al (Bhushan et al., 2020). Overnight cultures were harvested by centrifugation at 8000 × g for 10 min, and the pelleted cells were suspended in PBS (pH 7.2). Cell suspensions standardized to approximately 10^9^ CFU/mL were then exposed to acidic environments (pH 1, 2, and 3) and MRS broth supplemented with varying concentrations of ox gall (0.3%, 0.5%, and 0.7%). The treatments were incubated at 37 °C for 0, 3 and 6 h. MRS broth without ox gall (pH 6.5) was used as a control. Then samples were progressively diluted and spread onto MRS agar plates to determine cell viability, expressed in colony-forming units per milliliter (CFU/mL).

##### 2.5.1.2 Tolerance to simulated gastrointestinal juices

Artificial GI fluids were formulated in accordance with the protocol described by Bao et al (Bao et al., 2010). For gastric juice, 0.35 g of pepsin was dissolved in 100 mL of 0.2% sterile saline, and the pH was adjusted to 2.5 using 1 mol/L HCl. The fluids were filtered using a 0.22 µm membrane filter. Artificial intestinal fluid was prepared by dissolving 0.9 g of trypsin and 1.8 g of bovine bile salts in 100 mL of distilled water containing 1.1 g of sodium bicarbonate and 0.2 g of sodium chloride. The pH of the solution was adjusted to 8.0 using 1 M sodium hydroxide. The prepared solution was then sterilized using a 0.22 µm membrane filter. Bacterial cultures were introduced into this synthetic intestinal fluid and viable cell enumeration were conducted by the plate count method at 0, 3 and 6 h post-inoculation.

##### 2.5.1.3 Phenol tolerance

To determine phenol tolerance, overnight-grown cultures of all seven isolates were inoculated into MRS broth supplemented with 0.4% phenol and conditioned at 37 °C for 24 h. Following incubation, viable cells were quantified using the plate count method (Hoque et al., 2010).

##### 2.5.1.4 Temperature and NaCl stress tolerance assessment

The procedure was optimized from the method documented by Abushelaibi et al. (2017) with slight amendments. Bacterial cultures nurtured for 24 h were placed into MRS broth and retained at varying temperatures (4, 25, 37, and 55 °C) for 24 h. Upon completion of incubation, aliquots were plated onto MRS agar and further maintained at 37 °C for 48 h. Similarly, 24 h cultures were seeded into MRS broth supplemented with sodium chloride at concentrations of 2, 4, and 6%, and incubated for 24 h. Cell viability under these constraints was determined using plate count assays.

### 2.6 Quantification of β-galactosidase production

Determination of β-galactosidase synthesis was performed following the technique detailed by Rai and Tamang (2022), with minor changes. Upon completion of the 24 h incubation period, bacterial cultures were pelleted by centrifugation at 12,000*×g* for 4 min at 4 °C. The obtained biomass was rinsed twice with PBS and resuspended in fresh MRS broth. One mL of the suspended cells was subjected to sonication for 5 min using an ultrasonic processor (frequency >20 kHz; Sonics Ultrasonicator, USA) to lyse the cells. The lysates were centrifuged, and 1 mL of the resulting supernatant was combined with 4 mL of o-nitrophenyl-β-D-galactopyranoside (ONPG, 4 mg/mL). The reaction mixtures were supplemented with 2% lactose and incubated at 37 °C over 24 h. After incubation, the aliquots were again centrifuged, rinsed twice with PBS, and reconstituted in the same buffer. The suspensions were then positioned in a 37 °C water bath for 15 min. To conclude, 0.5 mL of 1 M sodium carbonate was incorporated to terminate the reaction, and the absorbance was recorded at OD_560nm_. β-Galactosidase activity was quantified in terms of Miller Units, calculated as follows:


$$Miller\;Units(MU)=(OD_{420nm}/OD_{560nm}\times volume\times time)\times 1000.$$

Where volume = 1 mL and time = 15 min.

### 2.7 Extraction and quantification of EPS

#### 2.7.1 Extraction 

EPS extraction was carried out based on the protocol outlined by Rimada and Abraham (2003) with minimal changes. A 10 mL aliquot of the fermented culture was withdrawn and heated in a boiling water bath at 100 °C for 15 min to solubilize cell-bound polysaccharides and deactivate enzymatic activity. After cooling to room temperature, the suspension was centrifuged at 15,941 × g for 10 min at 20 °C to separate the biomass. To the resulting supernatant, 17 mL of 85% trichloroacetic acid (TCA) was added to a total volume of 100 mL, followed by incubation at 4 °C to facilitate protein precipitation. This was further subjected to centrifugation at 8,000 × g for 10 min. EPS was precipitated by adding ice-cold ethanol (−20 °C) in a 1:3 ratio. The mixture was stored at 4 °C for 48 h to complete precipitation; subsequently, it was centrifuged again (8,000 × g, 10 min, 4 °C). The collected pellet was dissolved in distilled water and considered the crude EPS extract.

#### 2.7.2 Quantification

To quantify the extracted EPS, a 5% phenol solution was prepared by dissolving 5 g of freshly weighed phenol crystals in distilled water and making up the volume to 100 mL. 400 µL of the EPS extract was pipetted into a clean test tube, followed by the addition of 400 µL of the 5% phenol solution. A blank was also set up using 400 µL of distilled water in place of the sample. Subsequently, 2 mL of concentrated sulfuric acid was rapidly added to each tube. The suspension was left undisturbed for 10 min, after which it was gently mixed and maintained at 30 °C for another 10 min. Reading was then measured at OD_490nm_, and the results were interpreted against the blank to determine the EPS concentration (Rimada and Abraham, 2003).

### 2.8 Evaluation of antioxidant potential

#### 2.8.1 Preparation of test sample

##### 2.8.1.1 Extracellular supernatant

The bacterial strains were propagated in MRS medium at 37 °C for 24 h until they reached the exponential phase. After incubation, cultures were subjected to centrifugation at 4,000*×g* for 10 min at 4 °C, and the cell free supernatants (CFS) were carefully harvested for subsequent analysis of DPPH activity.

##### 2.8.1.2 Intracellular extracts

The bacterial cells were rinsed thoroughly three times with PBS, harvested, and then disrupted using low-temperature ultrasonication (400 W, 5-sec pulses alternated with 5-sec pauses, repeated 80 cycles). Following disruption, the cell lysate was centrifuged at 8,000*×g* for 20 min, and the resulting supernatant was collected as the intracellular extract (ICE) for use in antioxidant activity evaluation.

#### 2.8.2 DPPH radical scavenging assay

The competence of the samples to eliminate DPPH radicals was assessed based on an adapted version of the procedure described by Fan et al (Fan et al., 2017). Briefly, 1 mL of ECS or ICE was combined with 1 mL of a 0.4 mM DPPH suspension prepared in ethanol. The mixture was left to stand in the dark at 37 °C for 30 min. After incubation, the absorbance was recorded at OD_517nm_. The percentage of radical scavenging activity was calculated using the equation below:


Scavenging Activity(%)=1−(A1−A2)/Ao×100

Ao = Absorbance of the control group (distilled water alone).

A1 = Absorbance of the test sample reacting with DPPH.

A2 = Absorbance of the blank group (absolute ethanol replacing the DPPH solution).

### 2.9 Elucidation of cell surface properties

#### 2.9.1 Cell surface hydrophobicity

Overnight bacterial cultures were harvested by centrifugation at 8,000*×g* for 10 min. The cell pellets were washed and resuspended in PBS, and the optical density of the suspension was adjusted and recorded at OD_560nm._ An equal volume (1:1) of hexane was added to 3 mL of this bacterial suspension, followed by vigorous vortexing for 2 min to facilitate interaction between the aqueous and organic phase (Abushelaibi et al., 2017). The mixture was allowed to rest undisturbed to enable phase separation at designated intervals (0, 3 and 6 h). Subsequently, the lower aqueous portion was delicately collected, and its absorbance levels were evaluated at OD_660nm_.


CSH(%)=[1−(A/Ao)]×100

Where A represents absorbance after phase segregation and Ao is the early absorbance.

#### 2.9.2 Auto-aggregation

This assay was performed according to the research designed by Abushelaibi et al (Abushelaibi et al., 2017). The strains were grown in MRS medium and centrifuged at 10,000*×g* for 15 min. The obtained pellets were purified twice and suspended in PBS. The aliquots were vortexed for 20–30 sec. The upper phase was mixed with 1.5 mL of PBS, and the absorbance was monitored at OD_600nm_ at 0, 3 and 6 h.

Auto-aggregation (%) = [1 - (At/Ao)] × 100, where A0 is the initial absorbance and At is the absorbance at the specified time t.

#### 2.9.3 Co-aggregation

To assess the co-aggregation ability of probiotic isolates with enteric pathogens, *S. flexneri* and *S.* Typhi were used as representative strains. Both the probiotic isolates and the test pathogens were grown overnight, retrieved by centrifugation at 10,000*×g* for 10 min, and rinsed thoroughly with PBS (Abushelaibi et al., 2017). The bacterial pellets obtained were suspended in PBS to standardize cell densities. Equal volumes (1:1) of the probiotic suspension and pathogen suspension were combined in sterile tubes and gently vortexed for 20–30 sec to ensure proper mixing. The mixtures were maintained at 37 °C, and co-aggregation was monitored by measuring the absorbance of the upper phase at OD_560nm_ after 0, 3 and 6 h.


Co−aggregation(%)=[1−(At/Ao)]×100.

Ao is the absorbance at the beginning of the experiment, and At is the absorbance at each time point t.

#### 2.9.4 Quantitative assessment of biofilm formation

Biofilm formation was identified as a desirable characteristic for probiotic strains, as it enhances binding to the gut barrier. The capacity of the bacterial isolates to form biofilms on abiotic surfaces was evaluated in well plates, adapting the procedure outlined by Bujnakova et al. (2014) with slight adjustments. After inoculation, the biofilm development was promoted by incubating the plates at 37 °C for 24 h. Post incubation, wells were carefully washed with sterile distilled water to remove non-adherent cells. Remaining attached cells were fixed and air-dried at ambient temperature. A 0.1% crystal violet (CV) solution was then added to each well to stain the biofilm. After 15 min, excess stain was discarded, and the wells were thoroughly rinsed under gentle running tap water to eliminate unbound dye. To quantify biofilm biomass, the retained stain was solubilized using glacial acetic acid, and the absorbance was recorded at OD_595nm_ using a microplate reader. Higher absorbance values corresponded to increased biofilm formation.

### 2.10 Safety assessment

#### 2.10.1 Evaluation of hemolytic activity

To assess potential hemolytic effects, freshly grown bacterial isolates were streaked onto blood agar plates supplemented with 5–10% defibrinated sheep blood and incubated at 37 °C for 24 h. After incubation, the plates were inspected for hemolysis around the colonies. Transparent zones surrounding the colonies indicated β-hemolysis (complete lysis of red blood cells), greenish discoloration represented α-hemolysis (partial lysis), while no change in the agar denoted γ-hemolysis (non-hemolytic). Only isolates exhibiting γ-hemolysis were considered non-virulent and retained for further probiotic evaluation (Halder et al., 2017).

#### 2.10.2 DNase activity

All seven strains were streaked onto DNase agar (Hi Media, Mumbai, India) plates utilizing 24 h cultures. DNase enzyme activity was indicated by the appearance of a clear halo surrounding the colonies after 48 h of incubation at 37 °C (Boricha et al., 2019). Methicillin-resistant *Staphylococcus aureus* ATCC 33591 (MRSA) was featured as a positive control.

#### 2.10.3 Antibiotic susceptibility test

Antibiotic susceptibility or resistance was evaluated using the Kirby-Bauer disk diffusion method, as documented by Bauer et al (Bauer et al., 1966). Bacterial isolates were cultured in MRS broth and incubated at 37 °C for 24 h. After incubation, using a sterile cotton swab distribute the bacterial suspension across the surface of Mueller-Hinton agar (MHA) plates. Five antibiotic discs (6 mm; Hi-Media, Mumbai, India) were selected for the assay The antibiotics included tobramycin (10 μg), vancomycin (10 μg), amikacin (10 μg), imipenem (10 μg) and imipenem (10 μg). The discs were gently placed onto the inoculated agar surfaces, and the plates were incubated at 37 °C for 24 h.

#### 2.10.4 Assessment of antimicrobial properties

The antimicrobial potential of the probiotic isolates was analysed against selected enteric pathogens, including *S. flexneri* (ATCC 12022), *S*. *sonnei* (ATCC 25931), S. Typhi (MTCC 733), *K*. *pneumoniae* (ATCC 700603), and E. *coli* (ATCC 49106), using well diffusion technique with slight changes from the method outlined by Gunyakti et al (Gunyakti and Asan-Ozusaglam, 2019). Pathogens were cultivated in LB broth and incubated at 37 °C for 18-24 h to reach the logarithmic growth phase. A bacterial suspension adjusted to approximately 1.5 × 10_8_ CFU/mL was aseptically distributed across LB agar plates using a sterile swab to ensure uniform coverage of the surface. Wells with a 6 mm diameter were aseptically created in the agar. Probiotic isolates were grown in MRS broth at 37 °C for 48 h, after which the cultures were centrifuged at 12,000*×g* for 20 min at 4 °C to collect the CFS. An aliquot of 100 μL of the CFS was carefully introduced into each well. The inoculated plates were incubated at 37 °C for 12-18 h under aerobic conditions. Post-incubation, the zones of inhibition surrounding the wells were measured in mm.

### 2.11 Molecular characterization

#### 2.11.1 Amplification of 16S rRNA gene

DNA extraction from all selected microbial isolates (n = 7) was performed using the traditional phenol:chloroform:isoamyl alcohol (25:24:1) procedure, following the protocol outlined by Sambrook et al (Sambrook et al., 1989). The purified DNA was used as a template for amplification of the complete 16S rRNA gene using primers 27F (5′-AGAGTTTGATCCTGGCTCA-3′) and 1390R (5′-GACGGGCGGTGTGTACAA-3′). Each PCR reaction was prepared in a 50 μL volume containing 1× Taq buffer with MgCl_2_, 50 μM of each dNTP, 0.2 μM of each primer, 1 unit of Taq DNA polymerase, and approximately 100 ng of template DNA. The PCR protocol began with an initial denaturation step at 95 °C for 10 min, accompanied by 35 cycles of denaturation at 95 °C for 45 sec, primer annealing at 55 °C for 1 min, and an extension phase at 72 °C for 90 sec. A final elongation step was performed at 72 °C for 10 min. Amplification was achieved using a QIAamp 96 thermal cycler (Qiagen, USA). PCR products were analyzed by electrophoresis on a 1.5% agarose gel to confirm successful amplification. Post-amplification, sequencing was carried out using an Applied Biosystems™ 3730xl DNA Analyzer. Original sequence data were evaluated for quality, and low-confidence regions were trimmed. High-quality reads were assembled into contigs using the CAP3 sequence assembly program. The assembled sequences were then aligned and compared against reference 16S rRNA gene sequences retrieved from the NCBI GenBank database and analysed using the BLAST sequence alignment tool to determine taxonomic affiliations. Verified sequences were subsequently submitted to the NCBI GenBank for accession and public availability.

#### 2.11.2 Statistical analysis

All assays were independently repeated three times, and the data were presented as the mean ± standard deviation (SD). Statistical evaluation was carried out using GraphPad Prism software (version 5.0; GraphPad Software Inc., USA). One-factor and two-factor analyses of variance (ANOVA) were employed to compare differences among groups. A significance level of *p* < 0.05, *p* < 0.01, *p* < 0.0001 and non-significant (ns) was adopted as the significance level.

## 3 Results

### 3.1 Selection and purification of microbial isolates

Eighty bacterial strains were isolated from 15 palm nectar samples sourced from a local farm in Devakottai, Tamil Nadu. Out of these, 56 exhibited broad-spectrum antimicrobial activity against selected pathogens. Among them, seven strains demonstrated strong adhesion abilities and resilience under acidic, bile, phenolic, and gastrointestinal-mimicking environments. Therefore, only these seven selected strains were subjected to further characterization and analysis.

### 3.2 Phenotypic and biochemical profiling

In the present investigation, all the tested bacterial isolates were exhibited as Gram-positive and rod-shaped, except for PN 72, which was cocci-shaped. The isolates formed white, creamy colonies with a jelly-like surface on MRS agar plates and exhibited no pigmentation. Furthermore, all strains are catalase negative, non-spore forming, and non-motile. Carbohydrate fermentation profiles were assessed using the Hicarbo kit, demonstrating the strains capacity to utilize a range of carbohydrates (**Table 1**).

**Table 1:** Carbohydrate fermentation profiles of probiotic strains isolated from palm nectar.

Carbohydrates	PN 1	PN 21	PN 31	PN 35	PN 63	PN 67	PN 72
Lactose	+	+	+	+	+	+	–
Xylose	+	+	+	+	+	+	–
Maltose	+	+	+	+	+	+	+
Fructose	+	+	+	+	+	+	+
Dextrose	+	+	+	+	+	+	+
Galactose	+	+	+	+	+	+	–
Raffinose	+	+	+	+	+	+	–
Trehalose	+	+	+	+	+	+	+
Melibiose	+	+	+	+	+	+	–
Sucrose	+	+	+	+	+	+	+
L- arabinose	+	+	+	+	+	+	–
Mannose	+	+	+	+	+	+	+

‘+’ indicates positive fermentation, while ‘–’ indicates no fermentation.

### 3.3 Functional assessment of probiotic traits

### 3.3 Resistance of probiotics to stimulated GI conditions

#### 3.3.1 Tolerance to acidic and bile conditions

**Figure 1:**
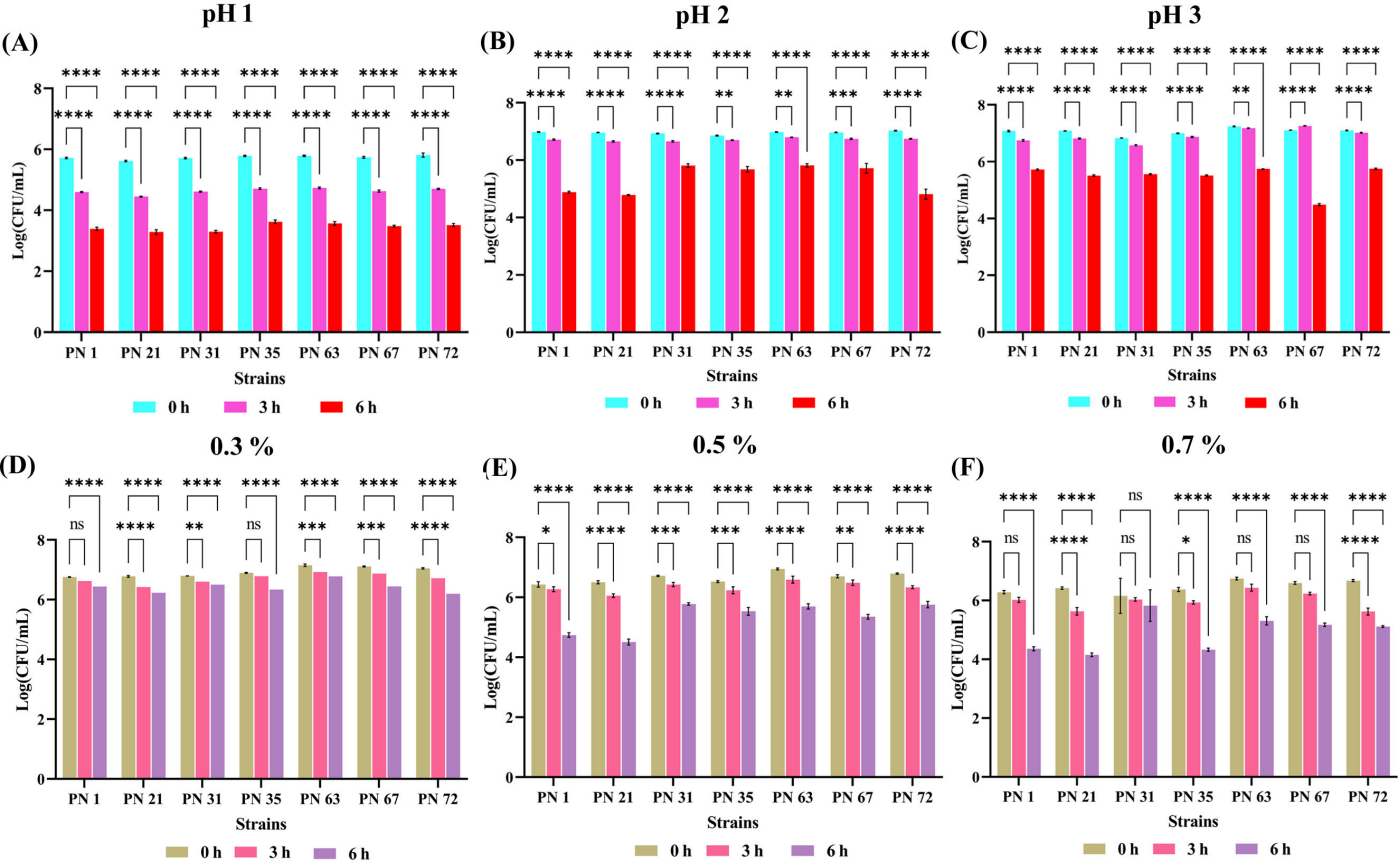
Acid and bile tolerance of seven probiotic isolates expressed as Log CFU/mL. Acid tolerance was evaluated at pH 1.0 **(A)**, pH 2.0 **(B)**, and pH 3.0 **(C)**, while bile salt tolerance was assessed at concentrations of 0.3% **(D)**, 0.5% **(E)**, and 0.7% **(F)**. Bacterial survival was measured at 0, 3, and 6 h of incubation at 37 °C. Results are presented as mean values ± standard deviation (SD) from three independent experiments. Statistical analysis was performed using two-way ANOVA; significant differences in survival rates (*p* < 0.05, *p* < 0.01, *p* < 0.0001 and ns) are denoted by an asterisk (*), (**), (****). Non-significant differences are marked as “ns”.

The acid tolerance of seven probiotic strains was evaluated at pH levels 1.0, 2.0, and 3.0 over incubation periods of 0, 3, and 6 h, as shown in **Figures 1A-C**. Viability was assessed by enumerating CFU, with results expressed as Log CFU/mL. All strains remained viable under highly acidic conditions, including pH 1.0 after 3 h of exposure, demonstrating notable acid resistance. A progressive, time-dependent decline in viable counts was observed across all pH levels, with variability among strains. After 6 h of incubation, moderate reductions in viability were recorded, yet all strains sustained survival. This consistent retention of viability, despite prolonged exposure, underscores the strain’s resilience to acidic environments. Overall, the low log reductions across conditions affirm the robust acid tolerance of these probiotic isolates.

The bile salt tolerance of seven probiotic strains was evaluated following exposure to ox gall bile salts at terminal concentrations of 0.3, 0.5 and 0.7% (w/v) over 0, 3, and 6 h, as depicted in **Figures 1D-F**. All strains exhibited substantial viability across all time points, including after 6 h at the highest concentration (0.7%), indicating strong bile resistance. A concentration- and time-dependent reduction in viable counts was observed, with increased bile levels exerting a more pronounced inhibitory effect.

#### 3.3.2 Tolerance to simulated gastrointestinal juices

**Figure 2:**
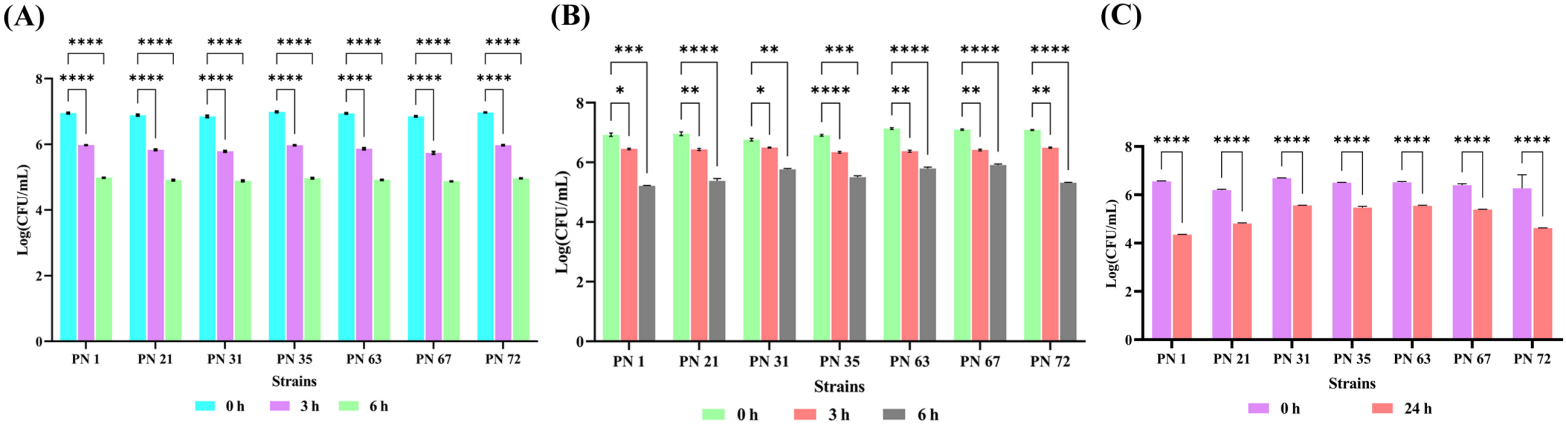
*In vitro* resistance of seven probiotic isolates to artificial gastrointestinal environment and phenol exposure, presented as Log CFU/mL. **(A)** Survival in mimicked gastric fluid and **(B)** simulated intestinal fluid was monitored at 0, 3, and 6 h of incubation at 37 °C. **(C)** Phenol tolerance was evaluated at 0 & 24 h of incubation at 37 °C. Data represent the mean ± standard deviation (SD) of three independent replicates. Statistical analysis was performed using two-way ANOVA; significant levels in survival rates (*p* < 0.05, *p* < 0.01, *p* < 0.0001) are denoted by an asterisk (*), (**) (****), respectively.

All seven isolates exhibited optimal growth under simulated gastric and intestinal environments. However, their viable cell counts significantly declined when exposed to simulated gastric juice at a low pH for an extended incubation period. The results for simulated gastric juice are presented in **Figure 2A**, showing that all isolates exhibited moderate survival at the end of 6 h. The impact of simulated intestinal juice is illustrated in **Figure 2B**, where all strains demonstrated the ability to survive in the intestinal fluid. Among them, PN 31 exhibited the highest survival rate over a 6-h period, though a gradual decline in growth was observed over time.

#### 3.3.3 Phenol tolerance

Phenol resistance serves as an important indicator of bacterial survival under intestinal conditions. As shown in **Figure 2C**, all tested isolates exhibited the ability to withstand phenol in the growth medium. Among them, PN 72 demonstrated the highest tolerance, with cell viability increasing from 6.26 to 6.62 Log CFU/mL over the incubation period. This was followed by PN 35 (6.50 Log CFU/mL), PN 63 (6.54 Log CFU/mL), and PN 67 (6.38 Log CFU/mL), which also exhibited notable resistance to phenol compared to the other isolates.

### 3.4 Temperature and NaCl stress tolerance assessment

**Figure 3:**
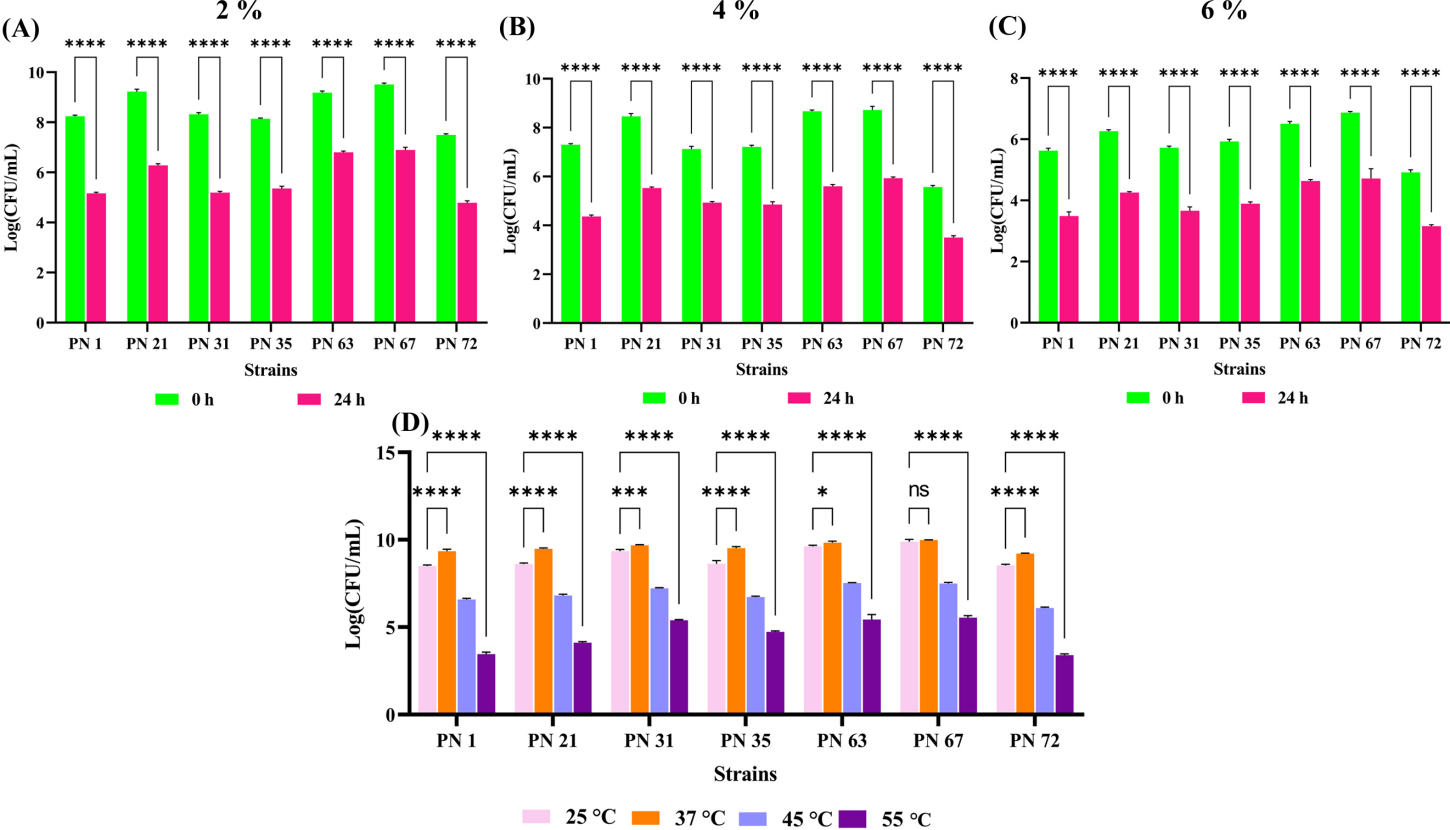
Effect of varying NaCl concentrations and incubation temperatures on the viability of seven probiotic isolates, expressed as Log CFU/mL. **(A-C)** Survival in 2%, 4%, and 6% NaCl concentrations, respectively, measured at 0 and 24 h. **(D)** Temperature tolerance assessed after 24 h of incubation at 25 °C, 37 °C, 45 °C, and 55 °C. Data are presented as mean ± standard deviation (SD) from three independent replicates. Statistical analysis was performed using two-way ANOVA; significant levels (*p* < 0.05, *p* < 0.0001) are indicated by asterisks (*), (****) and ns, ***p < 0.001.

The salt tolerance of the seven probiotic strains was systematically assessed at NaCl concentrations of 2%, 4%, and 6%, at both 0 and 24 h of incubation period, as presented in **Figures 3A-C**. The viability of the isolates declined with increasing salt concentrations. At 6% NaCl, all isolates exhibited a substantial decline in viability after 24 h exposure. Furthermore, the probiotic strains exhibited robust growth across all tested temperatures following 24 h of incubation (**Figure 3D**). Optimal growth was recorded at 25 °C and 37 °C for all isolates. In contrast, growth was significantly reduced at 55 °C, indicating a pronounced thermal sensitivity at elevated temperatures.

### 3.5 Quantification of β-galactosidase production

**Figure 4:**
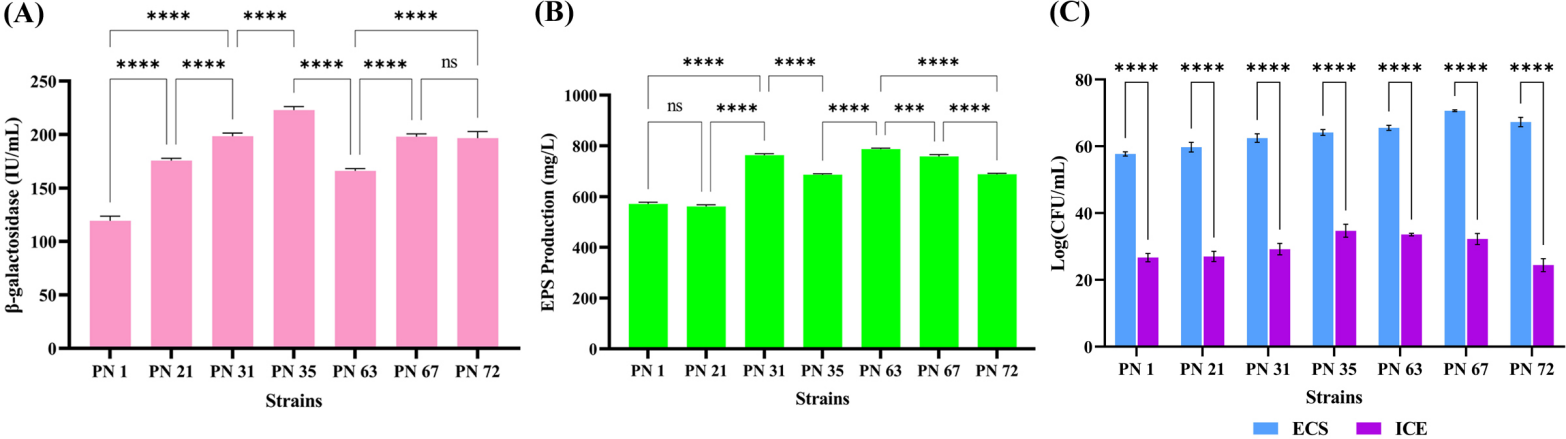
Functional properties of seven probiotic isolates. **(A)** β-galactosidase activity estimated after 24 h of incubation. **(B)** Exopolysaccharide (EPS) production by the isolates after 24 h **(C)** Antioxidant potential determined through DPPH radical scavenging activity of extracellular supernatant (ECS) and intracellular cell extract (ICE). Data are presented as mean ± standard deviation (SD) from three independent trials. Statistical significance was examined using one-way and two-way ANOVA, with significant differences (*p* < 0.0001) indicated by asterisks (****) and non-significant, ***p < 0.001.

The bacterial enzyme β-galactosidase plays a crucial role in enhancing lactose digestibility through hydrolysis. As shown in **Figure 4A**, maximum β-galactosidase activity was observed in the PN 35 (221.09 U/mL), followed by PN 31 (198.55 U/mL) and PN 72 (199.6 U/mL). Minimal activity was detected in PN 67 (197.45 U/mL). Enzyme production varied considerably among the seven bacterial strains examined. Notably, all screened isolates demonstrated substantial β-galactosidase activity, highlighting their potential role in alleviating lactose intolerance in the host.

### 3.6 Extraction and quantification of EPS

In this study, EPS production of seven isolates was quantitatively assessed. The highest EPS yield was observed in the PN 63 strain (785 mg/L), followed by PN 31 (763 mg/L) and PN 67 (756 mg/L). The PN 35 and PN 72 strains produced 691 mg/L and 681 mg/L of EPS, in that order, while PN 1 exhibited a slightly lower yield of 573 mg/L. Strain PN 21 showed the minimum EPS yield of 567 mg/L (**Figure 4B**). These findings indicate that the probiotic isolates evaluated in this study have a significant capacity for EPS production.

### 3.7 Evaluation of antioxidant potential

The antioxidant potential of the isolates, as measured by DPPH assay, is depicted in **Figure 4C**. Among the ECS samples, strain PN 67 exhibited the highest scavenging activity, reaching 70.66%. Notably, all other strains also demonstrated significant antioxidant potential, with scavenging rates exceeding 50%. In contrast, the ICE samples showed relatively lower DPPH scavenging activity. Among them, strain PN 35 recorded the highest activity at 34.70%. Overall, the ICE samples exhibited substantially lower scavenging capacities compared to the ECS samples and the standard antioxidant, ascorbic acid, which showed an 81.61% scavenging rate.

### 3.8 Elucidation of cell surface properties

#### 3.8.1 Cell surface hydrophobicity

**Figure 5:**
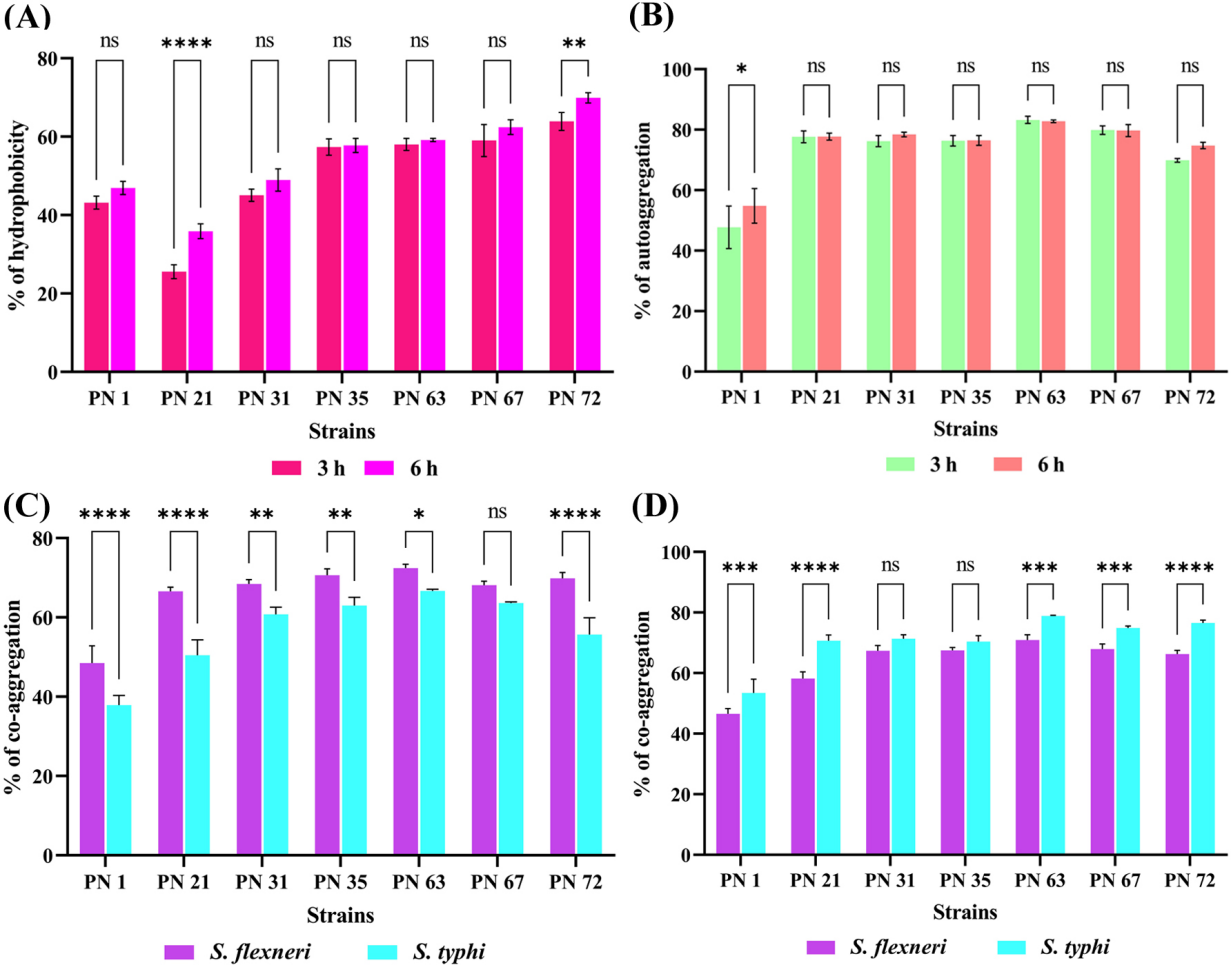
Cell surface properties of seven probiotic isolates evaluated at 3 and 6 h **(A)** Hydrophobicity percentage with hexane. **(B)** Auto-aggregation capacity. **(C, D)** co-aggregation of probiotic isolates with *S. flexneri* and *S.* Typhi at two different time intervals: **(C)** after 3 hours and **(D)** after 6 hours of incubation. Data are expressed as mean ± standard deviation (SD) from three independent replicates. Statistical relevance was assessed via two-way ANOVA; Variations were regarded as significant at *p* < 0.05 and *p* < 0.01, *p* < 0.0001 denoted by asterisks (**), (****) while non-significant differences are indicated by “ns”. * - p < 0.05.**** - p < 0.0001.

CSH plays a crucial role in non-specific interactions between bacterial cells and the host, influencing bacterial adhesion to mucosal surfaces. The interaction of bacterial cells with the non-polar solvent hexane was assessed to evaluate their Lewis acid-base surface properties. The results, presented in **Figure 5A**, reveal varying degrees of hydrophobicity among the seven selected isolates. PN 72 exhibited the highest adhesion to hexane, with values of 63.90 ± 2.28% at 3 h and 69.93 ± 1.29% at 6 h, indicating strong surface hydrophobicity. In contrast, PN 21 displayed the lowest adhesion, with values of 25.56 ± 1.74% at 3 h and 35.87 ± 1.87% at 6 h, suggesting a lower affinity for hydrophobic interactions.

#### 3.8.2 Auto-aggregation

Auto-aggregation capacity of seven isolates was assessed at 0, 3, and 6 h of incubation (**Figure 5B**). Auto-aggregation percentages ranged from 45% to 83%. Among the strains, PN 63 demonstrated the highest aggregation, reaching 83.25 ± 1.20% at 3 h and 82.76 ± 0.43% at 6 h, followed closely by PN 67 with 79.86 ± 1.39% at 3 h and 79.73 ± 1.99% at 6 h. In contrast, PN 1 exhibited the lowest auto-aggregation capacity, recording 45.27 ± 0.12% at 3 h and 59.29 ± 0.24% at 6 h. These results reveal considerable variability in the aggregation abilities among the strains, which may influence their potential probiotic efficacy.

#### 3.8.3 Co-aggregation

The co-aggregation interactions between seven probiotic isolates and selected human pathogens were examined in this study (**Figures 5C, D**). Among the isolates, strain PN63 demonstrated the highest co-aggregation with *S. flexneri*, reaching 72.43 ± 0.95% in 3 h and increasing to 78.85 ± 0.20% after 6 h of incubation. In contrast, strain PN1 exhibited the lowest co-aggregation, with values of 48.49 ± 4.34% at 3 h and 53.45 ± 4.53% at 6 h. Against *S.* Typhi, PN63 showed co-aggregation levels of 66.67 ± 0.41% after 3 h and 70.88 ± 1.74% after 6 h. Overall, all strains exhibited increased co-aggregation over time. These findings suggest that the probiotic strains, particularly PN 63, possess significant co-aggregation potential, which may potentially enhance their capacity to inhibit pathogen colonization.

#### 3.8.4 Quantitative assessment of biofilm formation

**Figure 6:**
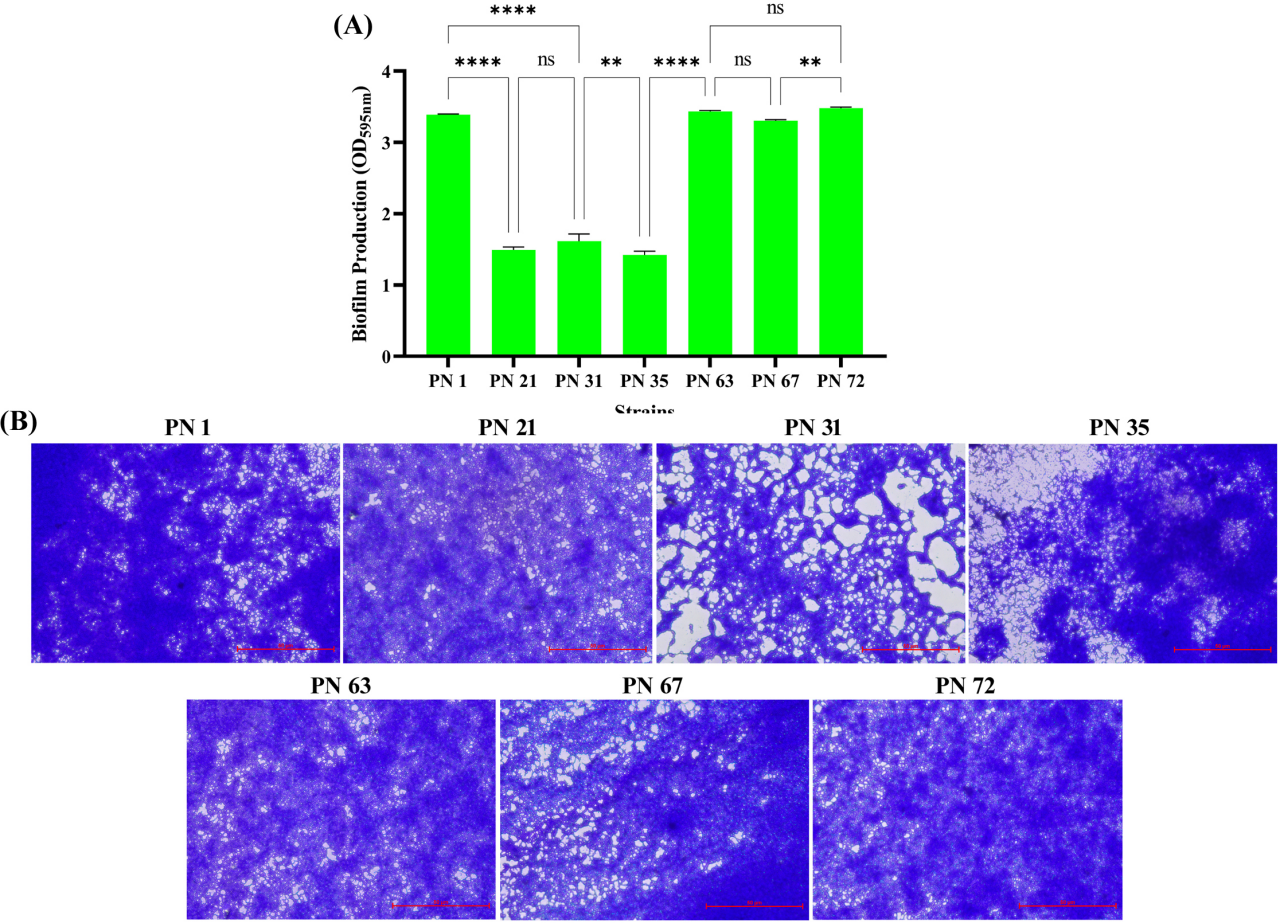
Biofilm production of seven probiotic isolates. **(A)** Quantitative assessment of biofilm formation measured by crystal violet staining at OD_595nm_. Values represent the mean ± SD based on three independent trials. Statistical outcome was evaluated by one-way ANOVA with multiple comparisons; differences are revealed as **** (*p* < 0.0001), ** (*p* < 0.01), and ns (not significant). **(B)** Representative light microscopy images of the stained biofilms formed by the seven isolates after 48 h incubation.

As illustrated in **Figure 6A**, all isolates exhibited strong biofilm formation. Notably, strains PN 31, PN 63, PN 67, and PN 72 demonstrated significantly higher biofilm-forming capacity compared to the others. Meanwhile, strains PN 1, PN 21, and PN 35 were classified as moderate biofilm producers. These observations were further validated through CV staining (**Figure 6B**), which confirmed the abundant biofilm formation on surfaces, as evidenced by microscopic analysis.

### 3.9 Safety assessment

#### 3.9.1 Evaluation of hemolytic activity

**Figure 7:**
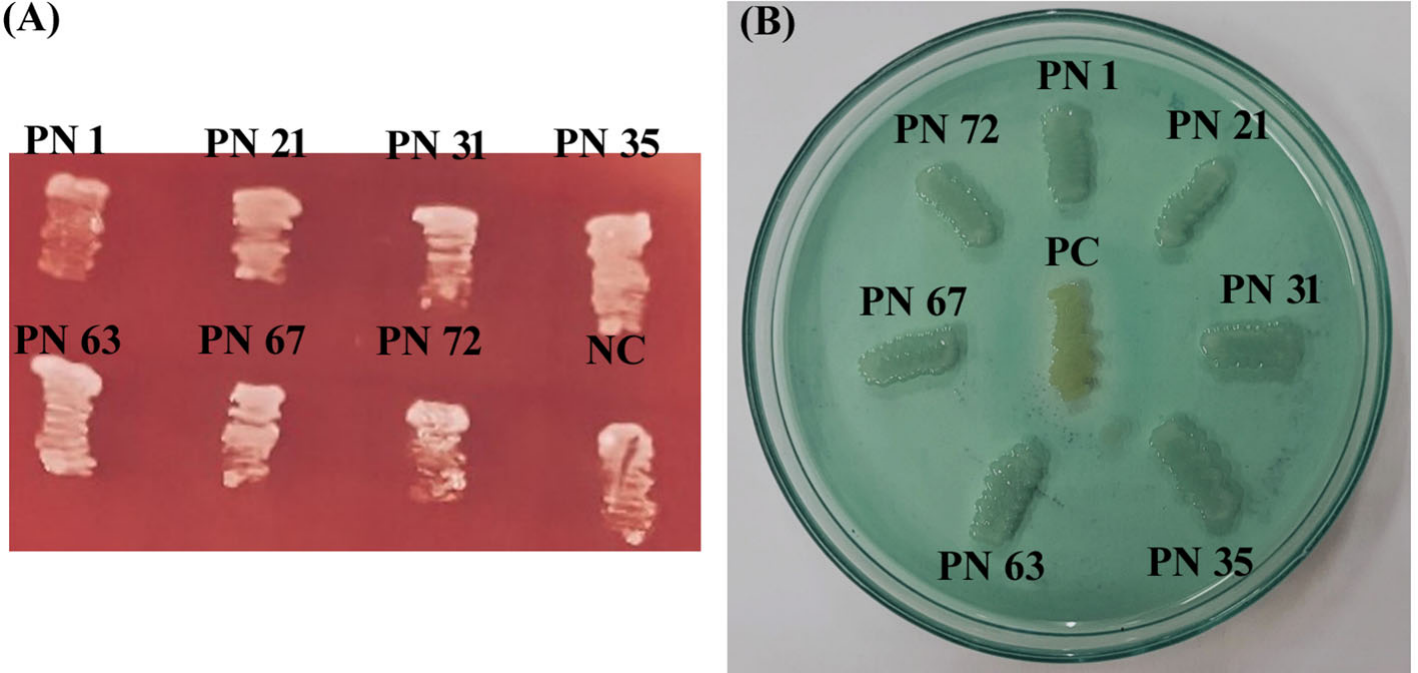
Safety assessment of seven probiotic isolates. **(A)** Hemolytic activity with a negative control (NC: *Escherichia coli* ATCC 49106) included for comparison. **(B)** DNase activity with a positive control (PC: *Staphylococcus aureus* ATCC 33591) used as reference. Results indicate the absence or presence of zone of hydrolysis (ZH).

In this investigation, none of the seven selected isolates demonstrated hemolytic activity on blood agar. After 24 h of holding period at 37 °C, no clear (β-hemolysis) or greenish (α-hemolysis) zones were observed around the colonies, indicating γ-hemolysis, showing no surrounding zone of lysis (**Figure 7A**). These results confirm that the isolates are non-virulent and safe for potential probiotic use. Lack of hemolytic activity represents an essential parameter for ensuring the safety of candidate probiotic strains.

#### 3.9.2 DNase activity

In the present study, isolates that did not exhibit any clear or pink halo zones on DNase agar upon completion of a 48-h incubation period at 37 °C were considered DNase-negative (**Figure 7B**). This absence of DNase activity suggests the non-pathogenic nature of these isolates. In contrast, the positive control MRSA produced a distinct clear zone around the colonies, confirming DNase production and validating the assay.

#### 3.9.3 Antibiotic susceptibility test

**Figure 8:**
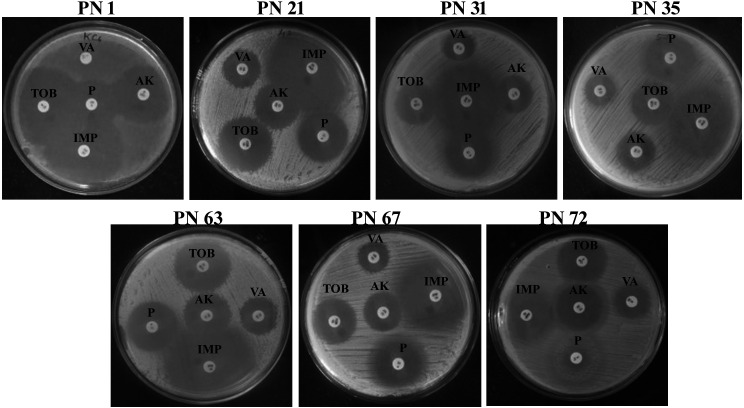
Antibiotic susceptibility of representative probiotic isolates determined using the disk diffusion method against various antibiotics. TOB, Tobramycin; VA, Vancomycin; AK, Amikacin; P, Penicillin; IPM, Imipenem.

In accordance with the recommendations of the European Food Safety Authority (EFSA), antibiotic susceptibility profiling was conducted for all seven selected isolates using antibiotics representing different drug classes. The inhibition zones were evaluated using the interpretive criteria outlined in the manufacturer’s reference chart. In the present investigation, all the evaluated isolates exhibited susceptibility to antibiotics such as amikacin, tobramycin, penicillin, vancomycin, and imipenem (**Figure 8**). However, strain PN 1 demonstrated resistance to vancomycin. These results indicate that the isolates possess minimal pathogenic traits and display unique strain-dependent features. Given their antibiotic susceptibility patterns, all seven isolates can be considered safe, reinforcing their suitability for potential probiotic applications. The variability in antibiotic sensitivity among the isolates is summarized in **Table 2**.

**Table 2:** Antibiotic susceptibility profiles of the seven probiotic isolates.

Strains	Zone of inhibition (mm)*				
	TOB (10 mcg)	VA (10 mcg)	AK (10 mcg)	P (10 mcg)	IPM (10 mcg)
PN 1	26	–	22	16	29
PN 21	25	15	22	28	35
PN 31	22	14	21	20	38
PN 35	23	14	21	22	35
PN 63	28	20	24	26	50
PN 67	35	23	30	35	51
PN 72	32	22	28	30	50

Results are shown as mean ± SD based on three independent trials. TOB, Tobramycin; VA, Vancomycin; AK, Amikacin; P, Penicillin; IPM, Imipenem. *Indicates that the values are shown as mean ± standard deviation (SD) based on three independent experiments.

#### 3.9.4 Assessment of antimicrobial properties

**Figure 9:**
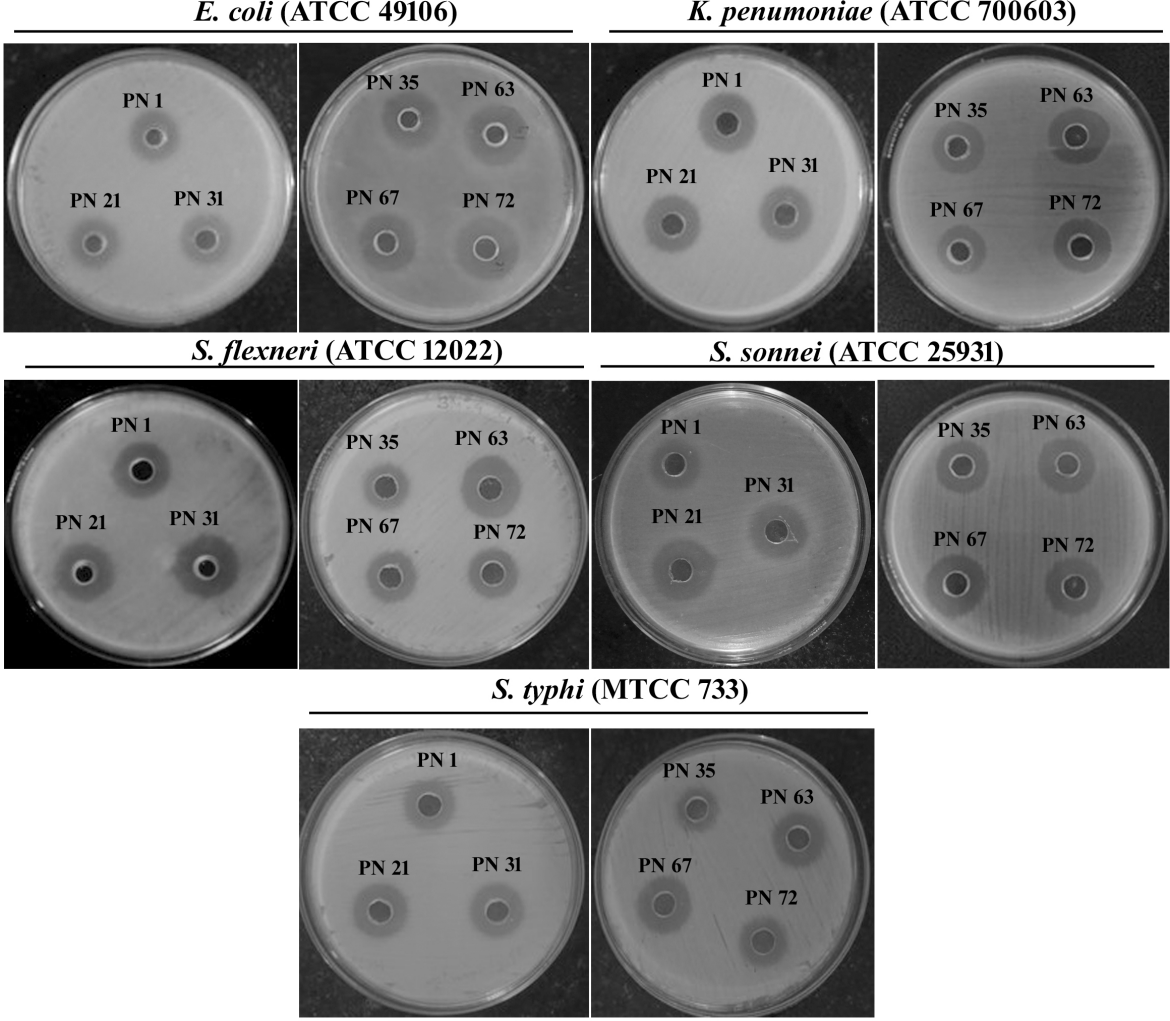
Antimicrobial activity of the cell-free supernatant from probiotic isolates against various enteric pathogens, evaluated using the agar well diffusion assay.

The inhibitory potential of the probiotic isolates against clinically relevant human pathogens is a key characteristic contributing to gut health maintenance. The inhibitory potential of the isolates was evaluated against five pathogenic microorganisms, as summarized in **Table 3**. The results revealed that the tested isolates exhibited significant antimicrobial effects, with varying degrees of inhibition against the target pathogens. Among the isolates, those demonstrating inhibition against all tested pathogens and exhibiting the largest zones of inhibition (ZOI) were considered the most effective (**Figure 9**). The supernatants of the probiotic strains displayed varying levels of antimicrobial activity against the indicator microorganisms, suggesting differences in the production of antimicrobial compounds.

**Table 3:** Antagonistic potential of probiotic isolates derived from palm nectar targeting five enteric pathogenic bacteria.

Strains	Zone of inhibition (mm)*				
	*S*. Typhi (MTCC 733)	*S. sonnei* (ATCC 25931)	*S. flexneri* (ATCC 12022)	*K. pneumoniae* (ATCC 700603)	*E. coli* (ATCC49106)
PN 1	18	15	17	17	17
PN 21	17	16	21	17	19
PN 31	21	19	20	22	17
PN 35	21	17	19	18	17
PN 63	22	19	21	20	17
PN 67	21	19	22	20	23
PN 72	20	19	20	21	20

Data represent mean ± SD from triplicate experiments. *Indicates that the values are shown as mean ± standard deviation (SD) based on three independent experiments.

### 3.10 Molecular characterization

#### Amplification of 16S rRNA sequences

**Figure 10:**
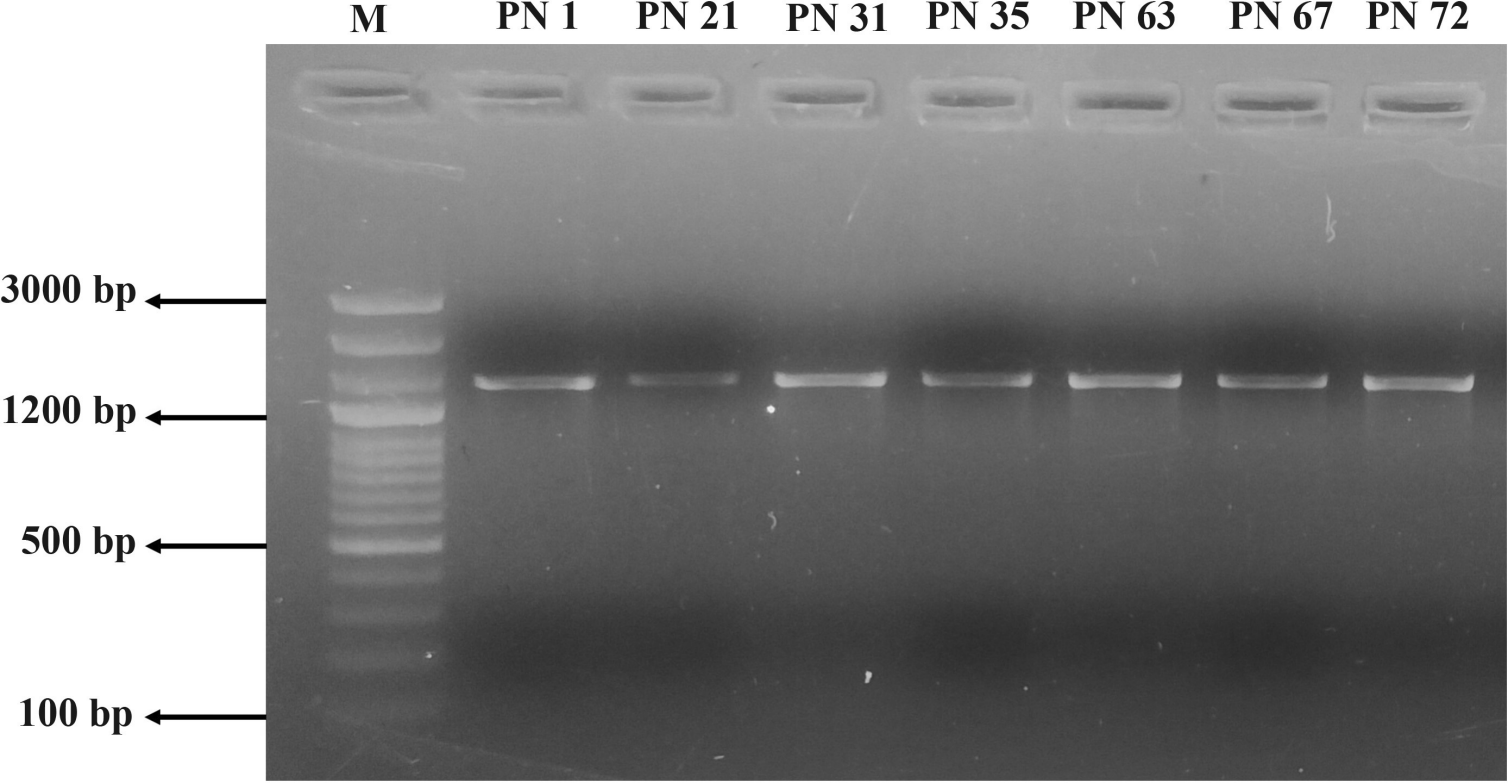
PCR amplification products of the isolated probiotic strains (PN 1, PN 21, PN 31, PN 35, PN 63, PN 67, and PN 72) visualized on a 2% agarose gel. Lane 1: DNA ladder (100 bp–3000 bp), lanes 2–8: PCR amplicons of the respective isolates.

Molecular identification of the seven most promising probiotic isolates was performed through 16S rRNA gene sequencing. All seven LAB strains yielded PCR amplicons of approximately 1500 bp, as confirmed by agarose gel electrophoresis (**Figure 10**). The retrieved 16S rRNA gene sequences were identified using the BLAST tool available at NCBI and later submitted to the GenBank repository (https://www.ncbi.nlm.nih.gov/genbank/), with accession numbers provided in **Table 4**. Sequence alignment revealed a high degree of similarity with previously reported 16S rRNA genetic sequences available in the GenBank database repository. Based on sequence homology, the isolates were identified as follows: PN1 - *Bacillus tequilensis*, PN21 - *Fructobacillus fructosus*, PN31 - *Fructobacillus parabroussonetiae*, PN35 - *Fructobacillus parabroussonetiae*, PN63 - *Fructobacillus fructosus*, PN67 - *Fructobacillus durionis*, and PN72 - *Staphylococcus hominis*.

**Table 4:** GenBank accession numbers of the seven identified probiotic isolates.

Strains	Genus/species	GenBank accession number
PN 1	*Bacillus tequilensis*	PV915462
PN 21	*Fructobacillus fructosus*	PV915463
PN 31	*Fructobacillus parabroussonetiae*	PV915464
PN 35	*Fructobacillus parabroussonetiae*	PV915465
PN 63	*Fructobacillus fructosus*	PV915466
PN 67	*Fructobacillus durionis*	PV915467
PN 72	*Staphylococcus hominis*	PV915468

## 4 Discussion

The present research was conducted to identify and evaluate bacterial candidates with robust probiotic traits. This research underscores the value of traditional fermented foods as rich yet underexplored reservoirs of probiotic microorganisms. Specifically, the study focused on the isolation of functional probiotic candidates from palm nectar (Pathaneer), a naturally fermented and widely consumed traditional beverage recognized for its nutritional value (Sornsenee et al., 2021). In this study, a total of 80 bacterial strains were isolated from fermented pathaneer, of which only seven demonstrated strong potential probiotic efficacies, including GI adaptability, adhesion ability, antioxidant activity, and antimicrobial properties. The objective of our research was not only to evaluate the probiotic attributes of these strains but also to examine their safety for prophylactic and therapeutic applications in the prevention of enteric infections.

The low pH in the stomach and the bile salts in the small intestine act as strong barriers to the survival of probiotic bacteria during digestion (Hsu et al., 2018). Acidic nature is essential for breaking down food, and it also creates a harsh environment for microbes. Similarly, bile salts in the intestine can damage bacterial cell membranes and reduce their survival in the gut (Oh and Jung, 2015). The ability to survive under these conditions depends on the bacterial strain and its defence mechanisms, such as acid tolerance and bile salt breakdown (Missaoui et al., 2019; Rodríguez-Sánchez et al., 2021). In this present investigation, seven strains recovered from palm nectar were examined for their acid and bile tolerance. While all strains showed some decrease in cell counts during prolonged exposure, they still upheld good probiotic potential, corresponding to previous findings where probiotics isolated from raw milk have been reported to withstand bile concentrations up to 0.3%, with viable counts ranging between 8.19 ± 0.01 and 9.21 ± 0.12 log CFU/mL after 3 h and 6 h of exposure (Reuben et al., 2020). Our isolates exhibited substantial resistance under similar stress conditions, with survival ranging from 3.40 ± 0.05 to 5.75 ± 0.03 log CFU/mL after 6 h and 6.53 ± 0.02 to 7.26 ± 0.01 log CFU/mL after 3 h in acidic conditions. Likewise, at 0.3% bile salt concentration, viable counts remained between 5.62 ± 0.12 and 6.92 ± 0.06 log CFU/mL after 3 h, and 4.15 ± 0.07 to 6.78 ± 0.08 log CFU/mL after 6 h of incubation (Reuben et al., 2020).

A further important trait of effective probiotics is their ability to survive the GI juices as they pass through to reach and colonize the host gut (Dunne et al., 2001). The acidic and basic environment of GI juices (pH 2.0 & pH 8.0) builds a major challenge for microbial survival, as it eliminates most ingested microbes (Vizoso Pinto, 2006). Consistent with previous reports, probiotic isolates exhibited notable tolerance to simulated GI conditions, with viable counts ranging from 3.00 ± 0.00 to 9.60 ± 0.06 log CFU/mL after 3 h and 7 h of incubation, respectively (Reuben et al., 2020). Similarly, in the present study, our isolates demonstrated strong survival under both gastric (4.98 ± 0.01 – 5.98 ± 0.02 log CFU/mL) and intestinal (5.21 ± 0.02 – 6.57 ± 0.06 log CFU/mL) conditions after 3 h and 6 h of incubation.

Phenolic compounds are toxic metabolites produced in the gut through bacterial deamination of amino acids derived from endogenous proteins, and they play a critical role in influencing microbial survival (Padmavathi et al., 2018). Previous studies have demonstrated that probiotic strains can exhibit enhanced tolerance to phenolic stress, with cell viability increasing from 66.50% to 88.91% after 24 h of exposure to 0.4% phenol (Zhang et al., 2022). In the present study, the isolates displayed even higher resilience, maintaining cell viability between 81.22% and 92.52% under comparable conditions. These findings suggest that the palm nectar-derived strains possess superior phenol tolerance, which may contribute to their persistence and functional stability within the gastrointestinal environment.

All the evaluated strains exhibited optimal growth at 25 °C and 37 °C after 24 h of incubation. The maximum cell densities were observed at 37 °C, ranging from 9.21 ± 0.03 to 9.98 ± 0.01 log CFU/mL. In contrast, a marked decline in growth was detected at higher temperatures, with viable counts decreasing to 6.59 ± 0.06 to 7.49 ± 0.07 log CFU/mL at 45 °C. This thermal sensitivity pattern aligns with the observations of Zang et al (Zhang et al., 2022). The isolates also tolerated NaCl concentrations of 2% and 4%, demonstrating good salt resistance. However, a marked decrease in growth was seen at 6% NaCl, indicating that higher salt levels adversely affect the viability of these strains.

Probiotic bacteria help mitigate lactose intolerance through two main mechanisms: by breaking down lactose in fermented foods during processing and by boosting β-galactosidase activity in the GI tract (Rai and Tamang, 2022). The present study showed that all isolated probiotic strains were found to produce β-galactosidase, though the enzyme levels varied noticeably among the strains. Our findings are consistent with those of Srinivas et al (Srinivash et al., 2023), who also reported strain-specific differences in β-galactosidase production, ranging from 196.4 to 217.9 U/mL. In our study, β-galactosidase activity ranged from 110.25 to 221.09 U/mL, indicating not only the presence of this key enzyme across all isolates but also highlighting the superior activity observed in certain strains. This variation underscores the potential of these palm nectar-derived probiotics for applications requiring efficient lactose hydrolysis and enhanced digestive support.

Furthermore, the probiotic strains examined in this study showed notable EPS production, which is valuable for both functional food applications and health benefits. EPS have been associated with health-promoting effects, including modulating immune responses, lowering blood pressure, reducing serum cholesterol (Shivangi et al., 2020) and even showing potential antitumor activity (Roux et al., 2022). Additionally, EPS can enhance bacterial adhesion to intestinal epithelial cells in a dose and strain specific manner, prevent pathogenic biofilm formation, and selectively stimulate the growth of other probiotic strains, thereby exhibiting prebiotic-like properties (Abushelaibi et al., 2017). Srinivas et al. reported that *Lactobacillus delbrueckii* strain GRIPUMSK produced EPS at a concentration of 690 mg/L (Srinivash et al., 2023). In contrast, our isolate PN 63 produced a higher yield of 785 mg/L, indicating its enhanced biosynthetic potential. These findings underscore the functional and therapeutic importance of EPS-producing strains in the development of probiotic formulations.

CSH and auto-aggregation analysis are important indicators of a probiotic strain’s ability to adhere to GI epithelial surfaces, which is crucial for preventing pathogen colonization through competitive exclusion and direct interaction (Sharma et al., 2021). Auto-aggregation refers to the ability of bacterial cells of the same species to cluster together, a trait closely linked to mucosal binding (Lukic et al., 2020). CSH, conversely, facilitates the initial non-specific attachment to host cells, which is further enhanced by specific bacterial surface molecules such as proteins and lipoteichoic acids (Tarique et al., 2022). Previous studies have reported that LAB species generally exhibit high auto-aggregation (>40%) and strong cell surface hydrophobicity (>60%) (Liu et al., 2022). In the present study, all isolates demonstrated strong auto-aggregation, with values ranging from 45% to 83%. Additionally, moderate hydrophobicity was observed, increasing over time and ranging from 25.56% to 69.93%. These results indicate a strong adhesive potential of the isolates, suggesting their capability to effectively colonize the gastrointestinal tract. Overall, our findings are consistent with earlier reports highlighting the importance of auto-aggregation and hydrophobicity in probiotic gut colonization (Somashekaraiah et al., 2019; Li et al., 2020; Jeong et al., 2021).

Co-aggregation is a critical defense mechanism through which probiotic strains block pathogen colonization. This process allows probiotics to physically bind with pathogenic bacteria, preventing them from attaching to host tissues (Choi et al., 2018; Nami et al., 2020). In the present study, we analysed the co-aggregation of the isolates with pathogenic bacteria, including *S.* Typhi MTCC 733 and *S. flexneri* ATCC 12022. Our results demonstrated that co-aggregation levels varied depending on the specific probiotic-pathogen combinations, indicating strain-dependent interaction patterns. The observed co-aggregation percentages ranged from 63.67% to 72.21% for certain combinations and 67.15% to 76.29% for others. These findings are in agreement with previous reports, which documented co-aggregation values ranging from 42.21% to 86.15%, and highlighted that co-aggregation is a selective trait influenced by the molecular composition of the strains and the type of pathogen involved (Nandha and Shukla, 2023). Overall, our results suggest that the tested probiotic isolates possess a substantial potential to interact with and possibly inhibit pathogenic bacteria through co-aggregation.

Biofilm formation is considered a beneficial trait in probiotic bacteria because it improves their ability to colonize mucosal layers and remain in the host for prolonged periods, thereby preventing pathogen colonization (Terraf et al., 2012). However, Sadeghi et al. (2022) demonstrated the capacity of *Lactobacillus* species to form biofilms, corroborating previous findings. In their study, the highest biofilm-forming values observed were 1.72 for *L. plantarum* S57, 1.63 for *L. paracasei* S23, 1.51 for *L. plantarum* S70, and 1.35 for *L. casei* S81, while the remaining strains exhibited values below 1. In contrast, our isolates displayed significantly stronger biofilm formation, with absorbance values exceeding 3.48 ± 0.02, highlighting their superior adhesive and colonization abilities. These results not only align with earlier observations but also extend current understanding by demonstrating exceptionally high biofilm-forming potential among isolates derived from palm nectar.

Bacterial cell surface components also contribute significantly to oxidative stress mitigation by neutralizing free radicals. The present study showed that the probiotic isolates exhibited strong antioxidant activity, particularly via DPPH radical scavenging. Among the different fractions tested, ECS cell extracts showed the highest DPPH scavenging potential, suggesting that secreted metabolites play a major role in antioxidant activity. This is partially aligned with (Wang et al., 2018), who reported that intracellular cell extracts of *Bifidobacterium* strains had higher DPPH and superoxide scavenging activity. Though the active fraction differed, both studies reinforce the strain-dependent nature of antioxidant capabilities in probiotics.

In addition to evaluating probiotic potential, this study also assessed the safety and biofunctional attributes of the chosen strains. All isolates were tested for hemolytic and DNase activity, antibiotic susceptibility, and antimicrobial activity. One isolate showed resistance to vancomycin, which is consistent with the observations of (Talib et al., 2019), who reported that LAB species generally exhibit intrinsic resistance to vancomycin and gentamicin. Importantly, this type of resistance is usually non-transmissible and is not regarded as a public health concern (Tokatlı et al., 2015). DNase activity testing revealed no DNase production, supporting the non-pathogenic nature of the isolates, since DNase is often associated with bacterial virulence (Kivanc et al., 2011). Finally, all isolates showed marked antimicrobial activity against common enteric pathogens, likely due to organic acid production during fermentation, which lowers environmental pH and disrupts pathogen metabolism. Collectively, these functional and safety attributes highlight the therapeutic promise of the isolated strains in promoting GI health.

## 5 Conclusion

In this study, seven potential probiotic strains were isolated from naturally fermented palm nectar and characterized for their gastrointestinal resilience, antimicrobial activity, and colonization potential. Among them, *Fructobacillus fructosus* PN63 and *Fructobacillus parabroussonetia* PN67 showed the most promising traits for surviving harsh GI conditions and adhering to intestinal surfaces. These findings highlight their potential application as functional probiotics in food or pharmaceutical formulations. Future studies focusing on *in vivo* evaluation, safety assessment, and mechanistic insights into host-microbe interactions will further establish their efficacy and pave the way for developing novel probiotic products.

## Data Availability

The datasets presented in this study can be found in online repositories. The names of the repository/repositories and accession number(s) can be found in the article/supplementary material.
